# Transcriptional Profiling of *Myceliophthora thermophila* on Galactose and Metabolic Engineering for Improved Galactose Utilization

**DOI:** 10.3389/fmicb.2021.664011

**Published:** 2021-04-28

**Authors:** Hanyu Wang, Tao Sun, Zhen Zhao, Shuying Gu, Qian Liu, Taju Wu, Depei Wang, Chaoguang Tian, Jingen Li

**Affiliations:** ^1^College of Biotechnology, Tianjin University of Science and Technology, Tianjin, China; ^2^National Technology Innovation Center of Synthetic Biology, Tianjin, China; ^3^Key Laboratory of Systems Microbial Biotechnology, Tianjin Institute of Industrial Biotechnology, Chinese Academy of Sciences, Tianjin, China

**Keywords:** *Myceliophthora*, galactose utilization, metabolic engineering, galactose transport, transcriptomic profiles

## Abstract

Efficient biological conversion of all sugars from lignocellulosic biomass is necessary for the cost-effective production of biofuels and commodity chemicals. Galactose is one of the most abundant sugar in many hemicelluloses, and it will be important to capture this carbon for an efficient bioconversion process of plant biomass. Thermophilic fungus *Myceliophthora thermophila* has been used as a cell factory to produce biochemicals directly from renewable polysaccharides. In this study, we draw out the two native galactose utilization pathways, including the Leloir pathway and oxido-reductive pathway, and identify the significance and contribution of them, through transcriptional profiling analysis of *M. thermophila* and its mutants on galactose. We find that galactokinase was necessary for galactose transporter expression, and disruption of *galK* resulted in decreased galactose utilization. Through metabolic engineering, both galactokinase deletion and galactose transporter overexpression can activate internal the oxido-reductive pathway and improve the consumption rate of galactose. Finally, the heterologous galactose-degradation pathway, De Ley–Doudoroff (DLD) pathway, was successfully integrated into *M. thermophila*, and the consumption rate of galactose in the engineered strain was increased by 57%. Our study focuses on metabolic engineering for accelerating galactose utilization in a thermophilic fungus that will be beneficial for the rational design of fungal strains to produce biofuels and biochemicals from a variety of feedstocks with abundant galactose.

## Introduction

The biological conversion of sugars from lignocellulosic biomass to fuels and commodity chemicals displays a promising and sustainable alternative to petroleum feedstock platforms (Lynd et al., 2002). Commercializing the production of biofuels from terrestrial plant biomass, including agricultural and forestry residues, has attracted considerable attention (Stephanopoulos, 2007; Tian et al., 2009). Marine algae, including macro-algae and microalgae, is also an attractive source for the production of biofuels. Production yields of marine algae biomass per unit area are much higher than those of terrestrial lignocellulosic biomass. Moreover, degradation of marine biomass is more easily compared with plant biomass due to the lack of recalcitrant lignin and cellulose crystalline structures (Wi et al., 2009; Wei et al., 2013; Noreen et al., 2016). Glucose and galactose present at 20 and 25% dry weight in marine macro-algae, respectively (Chi et al., 2012). It is necessary to utilize all sugars by the organisms for the cost-effective production of lignocellulosic-based products. Therefore, it is of great significance to explore the key pathways and regulation of microbial galactose metabolism.

Microbes use several pathways for galactose catabolism. Leloir (LL) pathway and De Ley–Doudoroff (DLD) pathway were common pathways and have been well-studied. The LL pathway mediates galactose utilization in bacteria, yeast, and filamentous fungi, such as *Escherichia coli*, *Saccharomyces cerevisiae*, and *Aspergillus nidulans* (Frey, 1996; Flipphi et al., 2009; Horvath et al., 2010; Lee et al., 2011). The LL pathway is involved in a total of four enzymes, and galactokinase is the key enzyme. Disruption of the gene encoding galactokinase in *E. coli* led to losing the ability to grow on galactose (Liu et al., 2014). In the LL pathway, galactose is phosphorylated to galactose-1-phosphate by galactokinase and then isomerized to glucose-1-phosphate by galactose-1-phosphate uridylyltransferase, accompanied by generation of uridine diphosphate (UDP)-galactose. UDP-galactose can then be recycled to UDP-glucose by UDP-galactose-4-epimerase. Phosphoglucomutase (PGM2) converts glucose-1-phosphate into glucose-6-phosphate, which enters the central glycolytic pathway and is ultimately converted to pyruvate.

The DLD pathway, similar to the Entner–Doudoroff (ED) pathway for glucose catabolism in archaea (Siebers and Schonheit, 2005), is found in some bacteria, such as *Azotobacter vinelandii* and *Caulobacter crescentus* (Kurn et al., 1978; Wong and Yao, 1994). In the DLD pathway, galactose is first oxidized to galactonate by galactose dehydrogenase and followed by D-galactonate dehydration to form 2-dehydro-3-deoxy-D-galactonate (KDG). Furthermore, KDG is phosphorylated by kinase and cleaved to release pyruvate and glyceraldehyde-3-phosphate by 2-keto-3-deoxygluconate (KDG) aldolase. The DLD pathway, requiring four enzymes, is a short and simple route of galactose catabolism and has been introduced into organisms to enhance galactose metabolism (Peabody et al., 2019).

In filamentous fungi, including *A. nidulans*, *Aspergillus niger*, and *Trichoderma reesei*, the oxido-reductive pathway is possessed to catabolize galactose (Fekete et al., 2004; Seiboth et al., 2007; Mojzita et al., 2012a, b), wherein galactose is converted to galactitol and catalyzed to L-sorbose. Subsequently, L-sorbose is reduced to D-sorbitol, which is converted to D-fructose and D-fructose-1-phosphate in turn. Moreover, enzymes that catalyze particular steps of the oxido-reductive pathway were distinct in fungi (Kowalczyk et al., 2015). The oxido-reductive pathway generally involves several enzymes of the pentose phosphate pathway and is regulated by XlnR and AraA in *T. reesei*, GalX in *A. niger*, and GalR, AraR, and XlnR in *A. nidulans* (Gruben et al., 2012; Kowalczyk et al., 2015; Benocci et al., 2018). Engineering regulation network has been considered as one approach for improving galactose fermentation.

The thermophilic filamentous fungus *Myceliophthora thermophila* (synonym *Thermothelomyces thermophilus*), which can secret a large number of hydrolytic enzymes and grow robustly on cellulosic materials, is exceptionally attractive for biorefinery construction (Berka et al., 2011; Singh, 2016). A suite of molecular biology tools, including the CRISPR/Cas9 method (Liu et al., 2017), is available for *M. thermophila*, which allows rational genetic engineering for fungal wider biotechnological exploitation. It has been developed into a mature system for carbohydrate hydrolase production at the industrial level (Visser et al., 2011). Recently, *M. thermophila* was metabolic engineered to produce malic acid by directly converting hemicellulose, cellulose, and raw corncob without adding extra hydrolase (Li et al., 2019). However, the efficiency of galactose utilization in *M. thermophila* is much less compared with other components of plant biomass, such as glucose, xylose, and arabinose. It will be important to capture this carbon for an efficient bioconversion process. To improve the galactose utilization in this thermophilic fungus, we performed the transcriptional profiling of *M. thermophila* on galactose to understand the native galactose utilization pathways. The oxido-reductive pathway for galactose breakdown was identified then, and the role of galactokinase in the LL pathway was assayed. Finally, heterologous galactose utilization pathway, DLD pathway, was introduced into *M. thermophila*, resulting in an engineered strain with 57% increase of galactose utilization. This study sheds new light on galactose utilization and metabolic engineering in filamentous fungi to efficiently convert all sugars in plant biomass into chemicals in future biorefinery.

## Materials and Methods

### Strains and Culture Conditions

*M. thermophila* ATCC42464 and its mutants (Table 1) were propagated on 1 × Vogel’s minimal medium (VMM) plates supplemented with 2% glucose at 35°C to obtain conidia after 8 days, and the corresponding antibiotic was added when needed for transformant screening.

**TABLE 1:** Fungal strains used in this study.

**Strains**	**Description**	**Source**
*M. thermophile* ATCC 42464	Wild-type	ATCC
OEgal2	Wild-type, overexpressing *gal-2*	This study
OEgal2Δ*galK*	OEgal2, Δ*galK*	This study
Δ*galK*	Wild-type, Δ*galK*	This study
HW2212	OEgal2, overexpressing *Ssgaldh*	This study
HW2302	OEgal2, overexpressing *Psgaldh*	This study
HW2506	OEgal2, overexpressing *Psgaldh*, and *Angdh*	This study
HW2607	OEgal2, overexpressing *Psgaldh*, *Angdh*, and *Ankdg*	This study
HW2705	OEgal2, overexpressing *Psgaldh*, *Angdh*, *Ankdg*, and *Pfkdgk*	This study

*E. coli* Mach1-T1 was used for vector construction and was cultivated in Luria–Bertani medium with 100 μg/ml ampicillin or 50 μg/ml kanamycin for plasmid selection.

### Plasmid Construction

All primer sequences used in this study are listed in Supplementary Table 1, for the construction of plasmids overexpressing target genes. The strong constitutive promoter of *eif* (Mycth_2297659) was used to efficiently overexpress galactose transporter gene *gal-2* (GenBank: 850770) from *S. cerevisiae.* The amplicons of P*eif* and *gal-2* were ligated between the *Bgl*II and *Bam*HI sites of pAN52-PgpdA-*bar* (Gu et al., 2018) to generate the corresponding plasmid P*eif*-gal2, using NEB Gibson Assembly Kit.

To convert D-galactose to galactonate, codon-optimized galactose dehydrogenase-encoding genes *Ssgaldh* (GenBank: AEY70388) from *Sulfolobus* sp. and *Psgaldh* (GenBank: PBP91902.1) from *Pseudomonas syringae* were controlled under strong constitutive promoter P*ap* from plasmid pPK2BarGFP. D-Galactonate dehydratase (GenBank: CAK43236.1) from *A. niger* catalyzes the dehydration of D-galactonate, and the open reading frame was codon-optimized, artificially synthesized, and controlled by promoter P*pdc*. Artificially synthesized genes encoding 2-keto-3-deoxygalactonate (KDG) aldolase AnKdg (GenBank: ABQ53586.1) from *A. niger* and KDG kinase PfKdgk (GenBank: QJP96888.1) from *Pseudomonas fluorescens* were regulated by the constitutive promoters P*cyc* and P*tef*, respectively. In addition, the artificially synthesized genes in this study, including *Psgaldh*, *Angdh*, *Ankdg*, and *Pfkdgk*, were codon-optimized on the basis of *Neurospora crassa* codon frequency^1^.

Plasmid for single guide RNA (sgRNA) expression was constructed as described previously (Liu et al., 2017). Briefly, the sgRNACas9 tool (Xie et al., 2014) was used for identifying specific sgRNA target sites in target genes (*galK*, Mycth_2299590) with the *M. thermophila* genome sequence and target gene sequences as the input. Oligos with no off-target probability were selected. The *M. thermophila* U6 promoter and a target-directed sgRNA fragment were amplified from the U6p-sgRNA plasmid (Liu et al., 2017) and cloned into blunt cloning vector pJET1.2.

For constructing the vector-carrying donor DNA for disrupting *galK*, the 5’- and 3’-flanking fragments of *galK* were amplified from the *M. thermophila* genome, fused with P*trpC*-*neo* from plasmid p0380-neo (Xu et al., 2015), and cloned into pPK2BarGFP digested by *Xba*I and *Eco*RV to obtain the donor DNA sequence donor-gGalk-neo.

All vectors were constructed using *E. coli* Mach1-T1, and the target genes cloned into shuttle vectors were sequenced to verify the authenticity of the plasmid construction.

### *Myceliophthora* Transformation

There was polyethylene glycol-mediated transformation of *M. thermophila* protoplasts (Liu et al., 2017). For gene overexpression, 10 μg of linearized plasmid was transformed into *M. thermophila* protoplasts. Putative transformants were selected on agar plates supplemented with corresponding antibiotics and confirmed *via* PCR amplification with paired primers. For gene disruption, sgRNA and donor expression cassette for *galK* disruption and Cas9-expression PCR cassette were mixed and co-transformed into *M. thermophila* wild-type strain and OEgal2 strain.

Putative transformants were selected with corresponding antibiotics (90 μg/ml G418 or 90 μg/ml PPT), followed by sequential identification *via* PCR with paired primers.

### Analysis of Galactose Consumption Rate

Strains were incubated in 100-ml VMM supplemented with 2% glucose at 45°C for 18–20 h and then washed three times with sterile water. Subsequently, the mycelia were transferred to Vogel’s salts containing 2% D-galactose for an additional 4 h. After that, the mycelia were washed three times and resuspended in uptake buffer (VMM, 10-mM D-galactose, and 10 μg/ml cycloheximide) for 20 min. The amount of residual sugar in the supernatant was determined, and the fungal biomass was completely dried to determine the dry weight for data normalization.

### Assay of Galactose Utilization

To evaluate the utilization rate of galactose, shaken-flask cultivation was performed in 100-ml VMM supplemented with 2% glucose in 250-ml Erlenmeyer flasks. Mature spores were inoculated with a final concentration of 2.5 × 10^5^ spores/ml, and the culture was incubated at 45°C with shaking at 150 rpm, and samples (1 ml) were taken at different intervals.

### Intracellular Metabolite Extraction

After pre-cultured in VMM with 2% glucose for 18 h, fungal mycelia were washed by sterile water and transferred into VMM containing 0.5% galactose for induction for an additional 4 h. Then, mycelia were collected and immediately homogenized in liquid nitrogen and ground into a powder in a prechilled mortar with a prechilled pestle. The paste was transferred into an l-ml extraction buffer (100-mM Tris pH 7.4). After centrifugation for 10 min at 4°C, the supernatant was used for intracellular production determination and protein qualification.

### Analytical Methods

Protein concentration in supernatants was measured using a Bio-Rad protein assay kit based on absorbance at 590 nm, using bovine serum albumin used as the standard.

Galactose, galactonate, and 2-keto-3-deoxygalactonate were determined by high-performance liquid chromatography using an instrument (e2695; Waters, Manchester, United Kingdom) equipped with an Aminex HPX-87H column (Bio-Rad) at 35°C and a Waters 2414 refractive index detector at 40°C. With a constant flow rate of 0.5 ml/min, 5-mM sulfuric acid was used as the mobile phase.

### Analysis of Cell Tolerance to Chemical and Environmental Stresses

Fungal sensitivities to oxidative stress, high osmotic stress, and cell wall disturbance were assayed by spreading 3-μl aliquots of conidial suspension containing 2.5 × 10^5^ spores onto agar plates alone or supplemented with hydrogen peroxide (H_2_O_2_; 1 mM), sodium chloride (NaCl; 0.5 M), or Calcofluor White (20 μg/ml). Plates were incubated at 35°C for 6 days.

### Transcriptomic Analysis

*M. thermophila* cultures were grown 18 h in VMM with 2% glucose and washed with sterile water. Mycelia were resuspended in 100-ml VMM with 2% carbon source (glucose or galactose) or no carbon source added. Total RNA was extracted with a modification of the method described previously, using TRIzol reagent (Tian et al., 2009). Genomic DNA contamination was eliminated by an additional clean-up using the RNeasy mini kit (Qiagen), according to the manufacturer’s RNA clear-up instructions. RNA integrity and concentration were determined using Nanodrop and agarose gel electrophoresis.

Complementary DNA libraries were prepared with standard protocols from Illumina and sequenced on an Illumina Novaseq platform to generate 150-bp paired-end reads. Independent duplicate cultures were sampled to avoid random error. The clean reads were mapped against predicted transcripts from the *M. thermophila* genome with less than two-base mismatching, using Tophat (v2.0.8b) (Trapnell et al., 2012). The alignment results were stored in SAM format files for subsequent analysis. The counts of reads that uniquely mapped to only one gene were calculated for each gene by Htseq-count^2^ using SAM files and genome annotation file as input. The normalized expression values [reads per kilobase per million mapped reads (RPKM)] for each gene were calculated by the number of uniquely mapped reads. Differential expression gene was determined by DESeq package (v1.5.1) (Anders and Huber, 2010) with raw counts of reads mapping to unique genes as input. Genes with DESeq *P* < 0.05 were considered to be expressed with a significant difference between conditions. Only genes with | log_2_ FoldChange| ≥ 1 and RPKM value ≥ 20 in at least one condition were considered for further analysis. The data were stored in Supplementary Table 2.

### Statistical Significance Tests

One-tailed homoscedastic (equal variance) *t*-test was used for adjusting statistical significance. n.s. indicates no statistical significance; ^∗^ represents a *p* < 0.05; ^∗∗^ represents a *p* < 0.01; and ^∗∗∗^ represents a *p* < 0.001.

## Results and Discussion

### Analysis of Transcriptional Profiling Predicted Out Native Galactose Utilization Pathways of *Myceliophthora thermophila*

**FIGURE 1:**
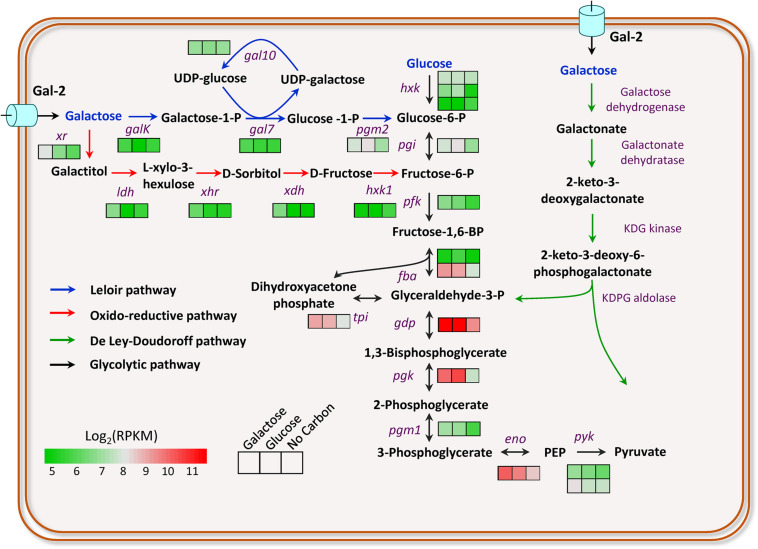
Transcriptional profiling of the genes involved in galactose breakdown in *M. thermophila*. Firebrick, highest expression; green, lowest expression. GalK, galactokinase; Gal7, galactose-1-phosphate uridyl transferase; Gal10, UDP-glucose-4-epimerase; Pgm2, phosphoglucomutase; Xr, xylose reductase; Ldh, L-arabinitol 4-dehydrogenase; Xhr, L-xylo-3-hexulose reductase; Xdh, xylitol dehydrogenase; Hxk1, Hexokinase-1. Detailed data were shown in Supplementary Table 3.

*M. thermophila* is capable of degrading lignocellulosic biomass and utilizing its hemicellulose components, including galactose. However, the taste of galactose to *M. thermophila* has not been investigated yet. Herein, to understand the galactose utilization of this thermophilic fungus, transcriptomic profiles of the cultures exposed to 2% galactose, 2% glucose, and no-carbon condition were performed. Based on transcriptomic data and bioinformatics analysis, the genes involved in galactose utilization were predicted out (Figure 1). The gene *galK* encoding galactokinase in the LL pathway showed an approximately 2-fold increased expression level on galactose, compared with that on glucose. However, the other three genes (*gal7*, *gal10*, and *pgm2*) in the LL pathway exhibited similar expression levels on galactose and glucose. Glucose-6-phosphate from galactose catabolism was converted to pyruvate *via* the glycolytic pathway. The expression levels of all glycolysis genes on galactose were similar to that on glucose (Figure 1). It has been reported that intermediates in glucose catabolism could induce carbon catabolite repression (Subtil and Boles, 2012). The gene (Mycth_2310085) encoding transcription factor CRE-1, a wide-domain master regulator of carbon metabolism, showed upregulated expression levels on galactose, compared with that on glucose. In yeast, Mycth_2310085 ortholog (Mig1) negatively regulates the expression of the galactokinase gene. In addition, one putative sugar transporter gene (Mycth_112491 with RPKM value > 100) was highly induced on galactose, and most likely, it is the major galactose transporter in this fungus, of which ortholog (NCU01633, *glt-1*) in *N. crassa* has been identified as a glucose transporter and also has the capability of transportation of multiple other monosaccharides, including galactose (Li et al., 2014).

The enzymes involved in the oxido-reductive pathway of galactose catabolism have been identified and characterized in *A. niger*, *A. nidulans*, and *T. reesei* (Fekete et al., 2004; Seiboth et al., 2007; Mojzita et al., 2012a, b). By BLAST search with protein sequences in *A. niger* as queries, the enzymes of this pathway in *M. thermophila* were predicted out (Figure 1), including xylose reductase (Xr, Mycth_43671), L-arabinitol-4-dehydrogenase (Ldh, Mycth_62052), L-xylo-3-hexulose reductase (Xhr, Mycth_2302811), xylitol dehydrogenase (Xdh, Mycth_2293953), and Hexokinase (Hxk1, Mycth_2306777) in turn. Xr and Xdh have been known previously involved in the catabolism of xylose and arabinose, whereas Ldh is also one of the enzymes in arabinose utilizing pathway. When responding to galactose, the genes (*xr*, *ldh*, *xhr*, and *xdh*) were expressed at significantly upregulated levels, compared with that on glucose and no-carbon conditions. The results suggested both the LL pathway and the oxido-reductive pathway contribute to galactose utilization in *M. thermophila*, of which the overall scheme was shown in Figure 1. The bioinformatics analysis and transcriptomic data clearly pull out the galactose utilization pathways in *M. thermophila* and have paved the way for improving galactose utilization through metabolic engineering in this fungus.

### Facilitating Galactose Catabolism by Introducing an Efficient Galactose Transporter

**FIGURE 2:**
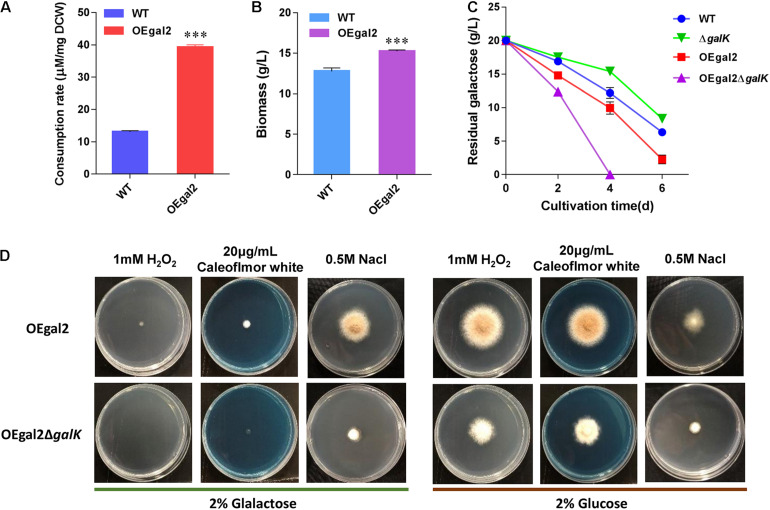
Physiological characterization of *M. thermophila* strains. **(A)** Assay of consumption rate of galactose. **(B)** Dry cell weight of culture of strain OEgal2 grown on galactose for 6 days. **(C)** Time course of galactose utilization in shaking flasks. **(D)** Sensitivities of the strain OEgal2Δ*galK* to oxidative stress, high osmotic stress, and cell wall disturbance. Mature spore was inoculated onto agar plates alone or supplemented with H_2_O_2_ (1 mM), NaCl (0.5 M), or Calcofluor White (20 μg/ml) and incubated at 35°C for 6 days. Values and error bars represent means and standard deviations of independent triplicate experiments, respectively.

Sugar uptake is the initial step for its utilization, and rapid substrate supply was recognized as a prerequisite for efficient cell factory production of biochemical (Ha et al., 2013; Lim et al., 2013; Hara et al., 2017). The previous report showing the galactose transporter Gal-2 from *S. cerevisiae* exhibits high galactose uptake activity (Kasahara and Kasahara, 2000). Therefore, to improve galactose utilization, the gene encoding galactose transporter Gal-2 driven by the strong constitutive promoter of *eif* (encoding elongation initial factor) was introduced into *M. thermophila* wild-type strain. After confirmation of the presence of the transgene by PCR analysis, the physiological characterizations of the resultant strain OEgal2 were conducted. When substrate consumption rate of strain OEgal2 was assayed, as we expected, an increase (3-fold) in the consumption rate of galactose was observed compared with that in the wild-type strain (Figure 2A).

To test the beneficial properties of Gal-2 overexpression on galactose utilization, the strain OEgal2 was incubated in VMM supplemented with 2% galactose. As shown in Figure 2C, an enhanced utilization rate of galactose was observed in strain OEgal2, compared with that of the parental strain. Consistently, dry cell weight of the strain OEgal2 showed a 19.7% increase than that of the wild-type strain after 6 days for fermentation (Figure 2B). The results clearly show that heterologous expression of galactose transporter does benefit galactose utilization in *M. thermophila*.

### Effect of the Disruption of Galactokinase on Galactose Utilization and Cell Stress Tolerance

Galactokinase is the key enzyme of the LL pathway, which catalyzes galactose to galactose 1-phosphate. To confirm the role of galactokinase in galactose utilization, galactokinase gene *galK* was knocked out in strains WT and OEgal2, *via* homologous replacement with a *neo*-inclusive cassette mediated by the CRISPR/Cas9 system (Liu et al., 2017) to generate the resultant strains △*galK* and OEgal2△*galK*, respectively. The correct recombination events in the resultant mutants were confirmed by PCR analysis. When physiological characterizations of the strains with deletion of *galK* on galactose were assayed, we found that the strains △*galK* and OEgal2△*galK* were still able to grow with galactose as the sole carbon source. It was suggested that galactose could be catabolized *via* an alternative degrading pathway—the oxide-reductive pathway. This phenomenon has been found in *A. nidulans* when the LL pathway was disrupted (Fekete et al., 2004). Nevertheless, the disruption of *galK* in the wild-type strain resulted in the decreased consumption rate of galactose. However, improvement of galactose utilization was observed in the strain OEgal2△*galK*, compared with that of the parental strain OEgal2 (Figure 2C). At the first 4 days of the culture, the rate of galactose utilization showed a 2-fold increase in the strain OEgal2△*galK* than that of the strain OEgal2. These observations might result from that the overexpression of galactose transporter complements reduced induction of galactose transporter gene by disrupting *galK*.

The LL pathway forms a link with the synthesis of cell wall components, lipopolysaccharides, and exopolysaccharides (Fekete et al., 2004; Lee et al., 2014). In addition, the activation of unfolded protein response in yeast is dependent on galactokinase activity (De-Souza et al., 2014). Therefore, we determined the effects of *galK* deletion on sensitivity to cell wall perturbation, oxidative stress, and high osmolality. As shown in Figure 2D, when grown on galactose and glucose, the strain OEgal2△*galK* showed increased sensitivity to Calcofluor White (20 μg/ml), which disrupts cell wall synthesis by binding to chitin (Ram and Klis, 2006). This observation suggested that the mutation of *galK* had altered cell wall integrity in *M. thermophila*. H_2_O_2_ is a common oxidative agent able to react with many biological molecules, including proteins and DNA. When responded to H_2_O_2_ (1 mM) under galactose or glucose condition, reduced growth phenotype of strain OEgal2△*galK* was observed, compared with the parental strain OEgal2, suggesting decreased tolerance to oxidative stress. Similarly, the strain OEgal2△*galK* grown on an agar plate supplemented with 0.5-M NaCl exhibited greater sensitivity to osmotic stress than the parental strain, indicating that the LL pathway in *M. thermophila* is involved in osmotic stress tolerance.

### Transcriptomic Analysis of *M. thermophila* With the Deletion of *galK* on Galactose

**FIGURE 3:**
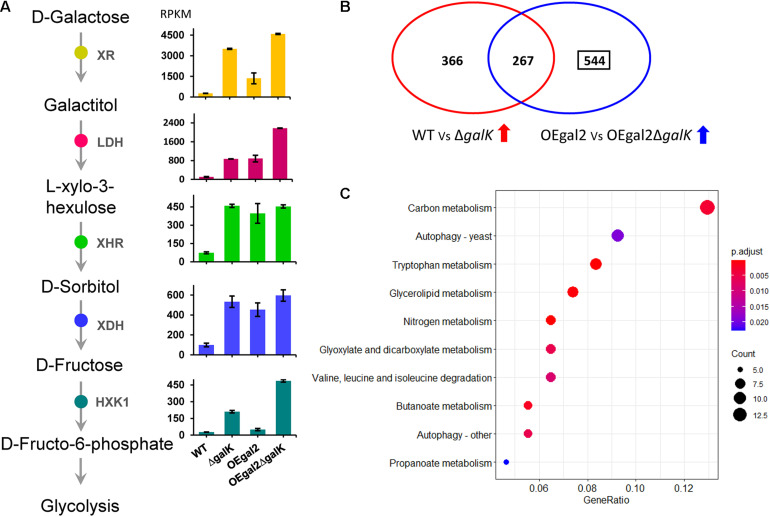
Transcriptional analysis of the strains *DeltagalK* and OEgal2*DeltagalK* when responding to galactose. **(A)** Expression levels of the genes involved in the oxide-reductive pathway. **(B)** Venn diagram of comparison of transcriptomes of *M. thermophila* strains grown on galactose. **(C)** Kyoto Encyclopedia of Genes and Genomes enrichment analysis of the 544 genes with upregulated expression levels in strain OEgal2*DeltagalK*. Detailed data were shown in Supplementary Table 7.

When exposed to galactose, the transcriptomic analysis indicated that the expression levels of genes *xr*, *ldh*, *xhr*, *xdh*, and *hxk1* were increased by 13.4-fold, 8.6-fold, 6.2-fold, 5.4-fold, and 7.8-fold, respectively, and 13-fold in the strain △*galK*, which verified that the oxido-reductive pathway was further activated, when the LL pathway was disrupted by the deletion of *galK* (Figure 3A). In addition, the gene Mycth_112491 encoding the predictive galactose transporter exhibited a significantly downregulated expression level (12-fold decrease) in strain △*galK* than that in strain WT, which might explain the reason for reduced galactose utilization in strain △*galK*. It has been reported that in *S. cerevisiae*, a genetic disorder in the galactokinase gene resulted in low uptake and catabolic rate of galactose (de Jongh et al., 2008). Moreover, compared with the wild-type strain, five genes involved in glycolysis, including phosphofructokinase, fructose-1,6-bisphosphate aldolase, triosephosphate isomerase, phosphoglycerate kinase, and enolase, displayed significantly reduced expression in the strain △*galK*. Expression levels of the four genes in the tricarboxylic acid cycle, including pyruvate dehydrogenase, succinate dehydrogenase, fumarase, and malate dehydrogenase, were decreased upon deletion of *galK*. Meanwhile, 17 genes were related to cell cycle and DNA processing in *M. thermophila*, of which 7 exhibited reduced expression in the strain △*galK*.

When the uptake rate of galactose was accelerated, the genes of the oxido-reductive pathway were induced at increased levels in strain OEgal2, compared with that of strain WT (Figure 3A). However, it was not observed for the LL pathway, suggesting that a complicated and comprehensive regulation network regulated the expression of genes involved in the LL pathway. Both disruption of *galK* and overexpression of galactose transporter gene *gal-2* resulted in improved galactose utilization of strain OEgal2△*galK*. To explain the observation, transcriptomic analysis of the strain OEgal2△*galK* on galactose was performed. Analysis of RNA-seq data from OEgal2△*galK* on galactose revealed a set of 811 genes that increased in expression levels and 950 genes that decreased in expression levels compared to data from strain OEgal2 (Supplementary Table 4). After subtracting 267 genes that were also differentially induced in strain △*galK* as compared to strain WT on galactose, functional enrichment analysis of the residual 544 genes was carried out using the Kyoto Encyclopedia of Genes and Genomes database (Figure 3 and Supplementary Tables 5, 6). The results revealed that the set of 544 genes was enriched in functional categories of carbon metabolism, nitrogen metabolism, tryptophan metabolism, glycerolipid metabolism, propanoate metabolism, pentose and glucuronate interconversions, and so on (Figure 3C). In terms of carbon metabolism, the genes involved in gluconeogenesis (phosphoenolpyruvate carboxykinase, Mycth_2315623; fructose-1,6-bisphosphatase, Mycth_2306943), glyoxylate cycle (isocitrate lyase, Mycth_110871 and Mycth_2311001; malate synthase, Mycth_2298071), and the tricarboxylic acid cycle (alpha-ketoglutarate dehydrogenase, Mycth_2312383) were included. In addition, compared with the strains WT, △*galK*, and OEgal2, the expression level of the gene *hxk1* in the strain OEgal2△*galK* was increased by 18-fold, 2.3-fold, and 9.7-fold, respectively, suggesting that hexokinase might be the rate-limiting enzyme of the oxido-reductive pathway in this thermophilic fungus.

### Heterologous Integration of Complete De Ley–Doudoroff Pathway Into *M. thermophila*

**FIGURE 4:**
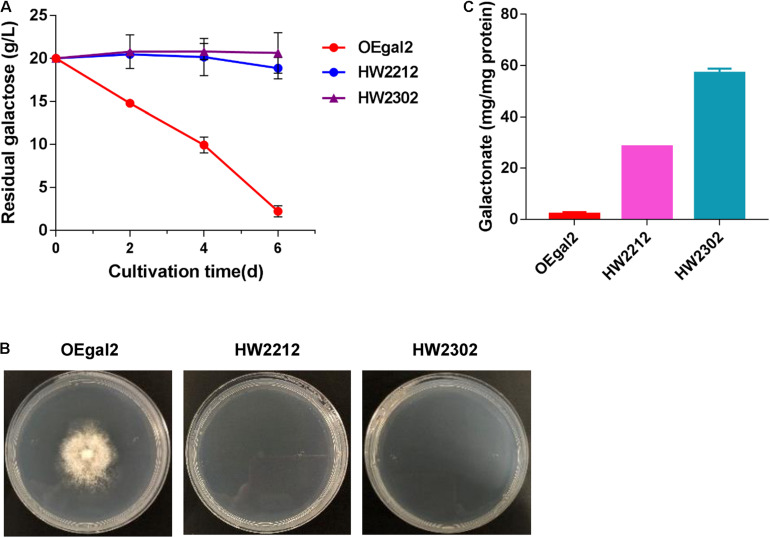
Physiological characterizations of *M. thermophila* strains HW2212 and HW2302 when grown on galactose. **(A)** Time profile of galactose utilization in shaking flasks. **(B)** Growth phenotype of the mutants on galactose for 6 days. **(C)** Assay of the accumulation of intracellular galactonate. Values and error bars represent means and standard deviations of independent triplicate experiments, respectively.

Introducing the whole heterogenous catabolic pathway has been considered an effective strategy for improving substrate fermentation. The DLD pathway, requiring only four enzymes to convert galactose to pyruvate, has been successfully introduced into the organism that does not natively utilize galactose and allowed for rapid growth with galactose as the sole carbon source (Peabody et al., 2019). Therefore, heterologous integration of the complete DLD pathway into *M. thermophila* was performed. A total of four enzymes, including galactose dehydrogenase, galactonate dehydratase, KDG kinase, and KDG(P) aldolase, were introduced one by one. To avoid great perturbation of the native galactose pathway, strain OEgal2 was chosen as the start strain. For the dehydrogenation of galactose to galactonate, two galactose dehydrogenase-encoding genes *Ssgaldh* from *Sulfolobus* sp. and *Psgaldh* from *P. syringae* have been characterized previously (Angelov et al., 2005; Azar et al., 2015) and were chosen to test in this fungus, generating the resultant strains HW2212 and HW2302, respectively. Both strains, HW2212 and HW2302, lost the ability to grow on galactose (Figures 4A,B). When intracellular metabolites were determined, the accumulation of galactonate in strains HW2212 and HW2302 was detected (Figure 4C), which might result in the incapability of growth on galactose. These results indicated that genes from archaea and bacteria were functionally expressed in this thermophilic filamentous. The strain HW2302 with the higher accumulation of galactonate was chosen for further engineering.

**FIGURE 5:**
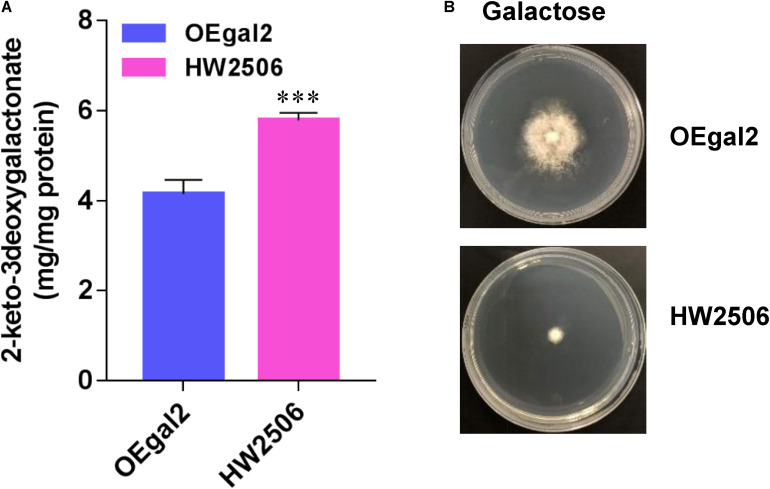
Physiological assays of strain HW2506 on galactose. **(A)** Accumulation of intracellular 2-keto-3deoxygalactonate. **(B)** Growth profiling of strain HW2506 on agar plate supplemented with 2% galactose after 6 d of incubation. Values and error bars represent means and standard deviations of independent triplicate experiments, respectively.

For conversion of galactonate to KDG, the gene *Angdh* encoding galactonate dehydratase from *A. niger* (Motter et al., 2014) was overexpressed in strain HW2302, generating the strain HW2506. When grown on galactose, strain HW2506 displayed serious growth defect, resulting from the higher accumulation of intermetabolite 2-dehydro-3-deoxy-D-galactonate (KDG) (Figure 5). In *A. niger*, KDG was cleaved into pyruvate and glyceraldehyde *via* 2-keto-3-deoxygalactonate (KDG) aldolase AnKdg and then catabolized (Elshafei and Abdel-Fatah, 2001). Therefore, the gene *AnKdg* from *A. niger* was first overexpressed in strain HW2506 to break down KDG, resulting in the strain HW2607. Unfortunately, when the growth phenotype of strain HW2607 was tested, the ability to grow on galactose was deprived. In microbes, glyceraldehyde was converted to glycerate by glyceraldehyde dehydrogenase (Reher and Schonheit, 2006). However, we found that glyceraldehyde dehydrogenase was not present in *M. thermophila*. Intracellular glyceraldehyde might be toxic to the fungus.

**FIGURE 6:**
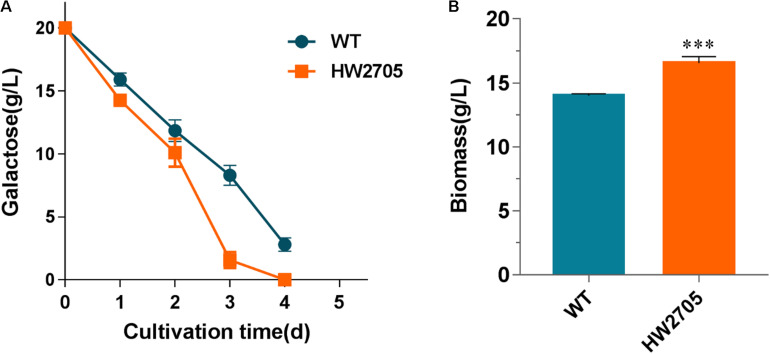
Growth phenotypes of strain HW2705 on galactose. **(A)** Time course of galactose consumption. **(B)** Dry cell weight of culture of strain HW2705 grown on galactose for 4 days. Pre-cultured mycelia on glucose were washed with sterile water and transferred into VMM containing 2% galactose for assays of sugar consumption and biomass formation in Erlenmeyer flask. Values and error bars represent means and standard deviations of independent triplicate experiments, respectively.

2-Keto-3-deoxygalactonate (KDG) aldolase has a wide substrate range, including KDG and 2-keto-3-deoxy-6-phosphogalactonate (KDGP). The genes *Pfkdgk* encoding KDG kinase from *P. fluorescens* have been successfully heterologously overexpressed for improved galactose utilization previously (Peabody et al., 2019). Herein, *Pfkdgk* was overexpressed in strain HW2607 to catalyze the phosphorylation reaction of KDG to KDGP before aldolase reactions. The end products were pyruvate and glyceraldehyde-3-phosphate. When incubated on a galactose medium, the resultant strain HW2705 showed an accelerated substrate utilization compared with the wild-type strain (Figure 6A). At the first 3 days of the culture, the rate of galactose utilization in strain HW2705 was increased by 57%. Consistently, cell dry weight of strain HW2705 showed an 18% increase than that of the wild-type strain (Figure 6B). These results indicated that the heterologous DLD pathway was functionally integrated and successfully improved galactose catabolism in *M. thermophila*.

## Conclusion

In this study, native galactose utilization pathways of *M. thermophila* were identified through transcriptomic analysis, and the oxido-reductive pathway for galactose catabolism was characterized firstly in this fungus. In addition, the effects of *galK* on galactose utilization and cell stress response were elucidated, which was necessary for the induction of galactose transporter. We also found that the LL pathway forms a link with the tolerance to cell wall perturbation, oxidative stress, and high osmolality. Moreover, simultaneous galactokinase disruption and overexpression of galactose transporter can improve galactose utilization significantly by the activation of the native oxido-reductive pathway. Finally, the heterologous galactose utilization pathway, the DLD pathway, was successfully integrated into *M. thermophila*, and the consumption rate of galactose was increased by 57% in engineered strain. This study deepens our understanding of galactose utilization in this industrial thermophilic fungus and provides novel strategies to improve hemicellulose-based chemical conversion in biorefinery using this thermophilic microbe.

## Data Availability Statement

The datasets presented in this study can be found in online repositories. The names of the repository/repositories and accession number(s) can be found in the article/Supplementary Material.

## Author Contributions

HW, JL, and CT conceived the project and wrote the manuscript. HW, TS, ZZ, SG, and TW performed the metabolic engineering experiments. JL, QL, and DW analyzed the data. All authors read and approved the final manuscript.

## Conflict of Interest

The authors declare that the research was conducted in the absence of any commercial or financial relationships that could be construed as a potential conflict of interest. The reviewer LL declared a shared affiliation, with the authors HW and DW to the handling editor at the time of the review.

## Footnotes

1 http://www.kazusa.or.jp/codon/

2 https://htseq.readthedocs.io/en/master/
