# Supplementary material for: Transcriptional Profiling of Myceliophthora thermophila on Galactose and Metabolic Engineering for Improved Galactose Utilization
Source: Front Microbiol. 2021 Apr 28;12:664011. doi: 10.3389/fmicb.2021.664011 (PMC8113861; doi:10.3389/fmicb.2021.664011)
Supplement: Supplementary file 2 [file Image_1.pdf]

Strains	Primers	PCR analysis
OEgal2	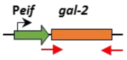	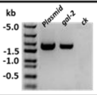
OEgal2Δ*galK*	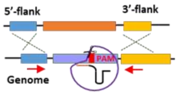	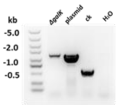
Δ*galK*	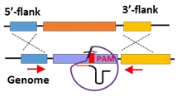	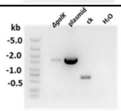
HW2212 and HW2302	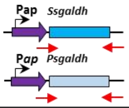	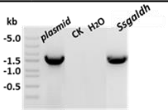 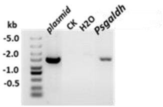
HW2506	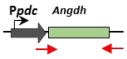	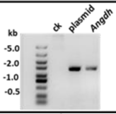
HW2607	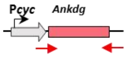	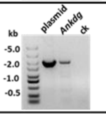
HW2705	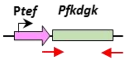	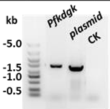

Fig. S1 PCR analysis of the mutants of *M. thermophila* generated in this study.
